# Endovascular treatment for 2 types of subclavian artery injury: A case report

**DOI:** 10.1097/MD.0000000000038892

**Published:** 2024-07-12

**Authors:** Gun Woo Kim, Suyeong Hwang, Kyoung Hoon Lim, Sung Hoon Cho

**Affiliations:** a Department of Surgery, Trauma Center, Kyungpook National University Hospital, School of Medicine, Kyungpook National University, Daegu, Korea.

**Keywords:** blung subclavian artery injury, endovascular treatment, penetrating subclavian artery injury, subclavian artery injury

## Abstract

**Rationale:**

Subclavian artery (SCA) injuries, though rare, carry significant morbidity and mortality risks due to significant blood loss causing hypovolemic shock. Early diagnosis and adequate treatment are crucial to minimize bleeding and associated morbidity. Recent advances in endovascular techniques offer faster and more accurate treatment options compared to traditional open surgical repair. This study demonstrates the efficacy of endovascular treatment in 2 cases of SCA injury and reviews its indications, limitations, and precautions.

**Patient concerns:**

A 69-year-old man presented with a penetrating SCA injury from a steel bar, and a 38-year-old woman presented with a blunt SCA injury caused by a fall. Both patients were hemodynamically unstable upon presentation.

**Diagnoses:**

Both patients were diagnosed with SCA injuries. The man had a penetrating injury, while the woman had a blunt injury, both resulting in hemodynamic instability and significant risk of hypovolemic shock.

**Interventions:**

Endovascular techniques, including the use of covered stent grafts, were employed to manage the injuries. These techniques allowed for rapid and efficient treatment, reducing the need for open surgical intervention.

**Outcomes:**

Both patients were successfully treated using endovascular methods and were discharged without any complications. The endovascular approach minimized blood loss, transfusion needs, and hospital stay.

**Lessons:**

This study demonstrates the effectiveness of endovascular techniques in rapidly diagnosing, bridging, and definitively treating SCA injuries, suggesting their use as a first-line therapy.

## 1. Introduction

The subclavian artery (SCA) is rarely injured during trauma (<5% of all vascular trauma)^[1–3]^; however, when it happens, it can cause devastating outcomes for trauma patients.^[4]^ A SCA injury can cause upper limb ischemia, brachial plexus injury, and even cerebral infarction from potential thromboembolism.^[5]^ In addition, this injury has a high mortality rate (ranging from 5% to 30%) due to massive blood loss causing hypovolemic shock.^[4,6,7]^ Therefore, early diagnosis and treatment are essential to improve mortality and morbidity rates. The classical management of SCA injury includes open surgical repair, which, however, is associated with significant morbidity because of the anatomical location of SCA.^[8]^ With recent advances in endovascular approaches, it is now possible to less invasively and promptly locate and treat SCA injuries.^[4,6,9,10]^ The purpose of this study is to demonstrate the efficacy and effectiveness of endovascular treatment through our experience in treating patients with SCA damage caused by 2 different mechanisms. This study also includes a review of the indications, limitations, and precautions of endovascular treatment.

## 2. Case presentation

### 2.1. Case 1

**Figure 1.**
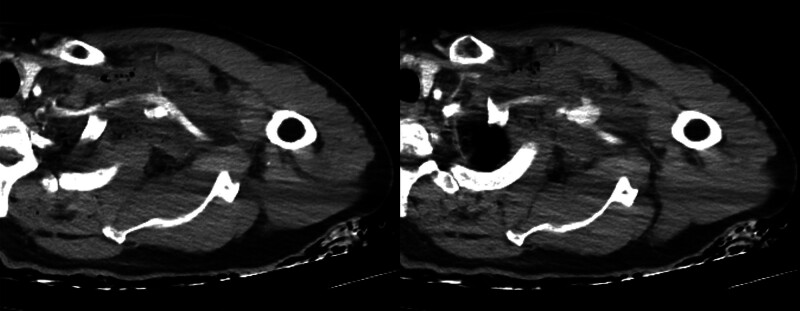
CT angiography showing extravasation of the left subclavian artery. CT = computed tomography.

A 69-year-old man sustained a penetrating wound from a steel bar piercing through his left axilla during construction work. On arrival, he had persistent bleeding from the axillary wound, and his systolic blood pressure (SBP) soon dropped below 50 mm Hg. His SBP recovered to 80 mm Hg with manual compression and a rapid transfusion. Computed tomographic (CT) angiography revealed an injury to the left SCA with extravasation and pseudoaneurysm (Fig. 1).

**Figure 2.**
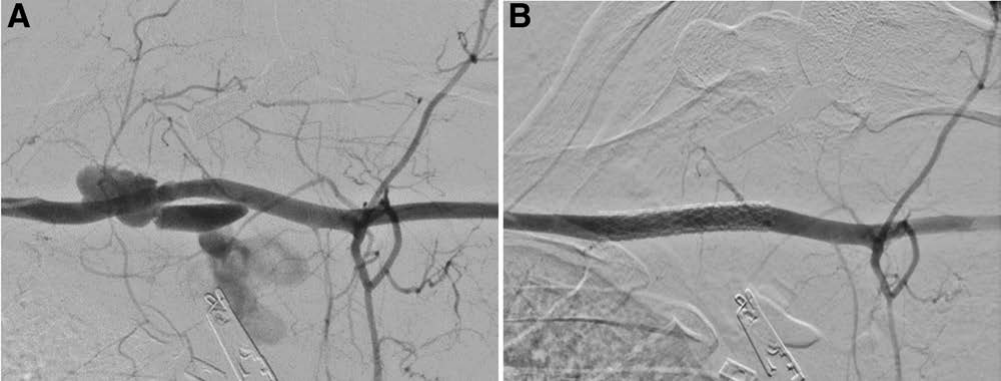
(A) Initial angiography showing extravasation of contrast at the left subclavian artery. (B) Completion angiography after the stent graft placement.

We decided to do endovascular repair with a covered stent graft because the injury site of the SCA was placed within the thoracic cage and was not angulated. The angiography was initiated within 90 minutes of arrival in the trauma resuscitation room. Through angiography, a stent graft (8.6 × 58 mm; LifeStream™ Balloon Expandable Vascular Covered Stent, BARD) was placed into the left SCA and adequate blood flow in the downstream artery was confirmed (Fig. 2A and B). Although we also identified an injury of the subclavian vein through a venogram, which was managed via compression because active venous bleeding was not seen and the flow of the collateral vein was good. The angiography and stent graft insertion took approximately 60 minutes, and 2 packs of red blood cells (RBC) and 3 packs of fresh frozen plasma (FFP) were transfused during the resuscitation and endovascular repair procedure. The patient was discharged after 17 days without any complications.

### 2.2. Case 2

**Figure 3.**
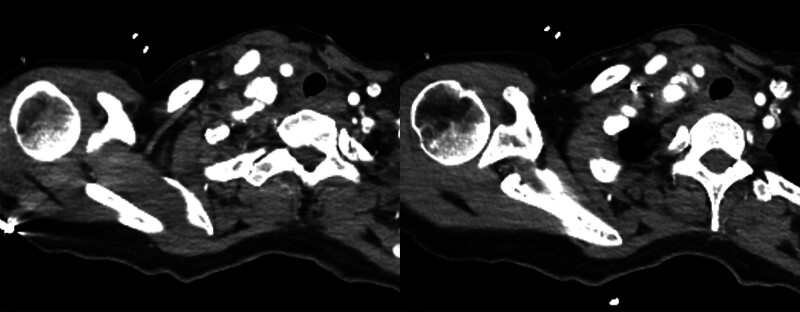
CT angiography showing extravasation of the right subclavian artery. CT = computed tomography.

A 38-year-old woman fell from the fifth floor. She was hemodynamically unstable, and her mentality was stuporous. A massive transfusion protocol (MTP) was performed to manage the blood loss, and a whole body CT scan was performed after she became hemodynamically stable. Contrast enhanced chest CT showed rupture of the right SCA with hemorrhage in the superior mediastinum (Fig. 3). Additionally, abdominal CT showed liver contusion, multiple vertebral fractures, intraperitoneal fluid collection, and free air around the duodenum suggestive of duodenal injury.

**Figure 4.**
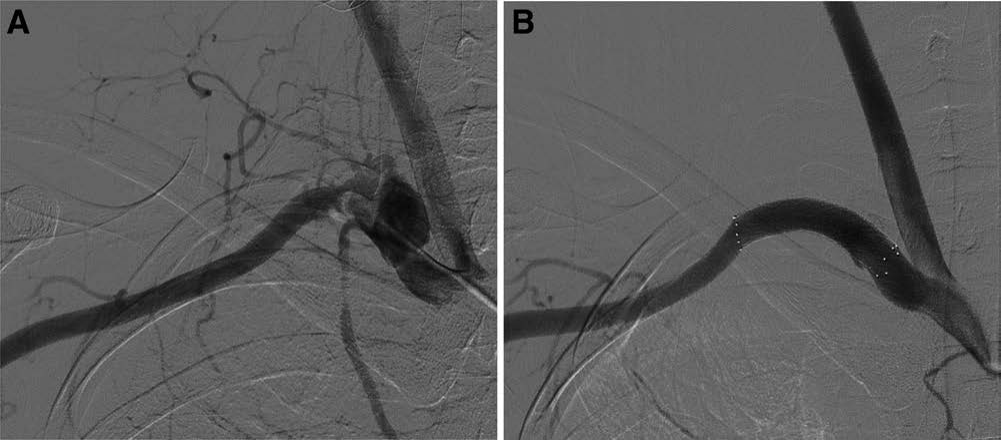
(A) Initial angiography showing extravasation of contrast at the right subclavian artery. (B) Completion angiography after the stent graft placement.

At first, we planned to insert a covered stent graft to stop bleeding from the perforated SCA and maintain the arterial flow in the right upper limb. Angiography was performed within 100 minutes of arriving in the trauma resuscitation room. Prior to stent graft insertion, we checked the flow on the other side of the vertebral artery to prevent the stent graft from covering the root of the vertebral artery and causing brain or spinal infarction; the left to right collateral flow in the vertebral artery was good. A covered stent graft (10 × 40 mm; Covera plus™ Vascular Covered Stent, BARD) was then placed into the right SCA (Fig. 4).

The complete procedure (angiography and stent graft insertion) took approximately 50 minutes, and 2 packs of RBC and 4 packs of FFP were transfused during resuscitation and intervention. The primary closure of the ruptured duodenum and repair of the jejunal serosal tear were performed by laparotomy right after the intervention. The patient’s right arm and function were preserved; she was discharged after 60 days of admission following additional operations.

## 3. Discussion

The SCA, well-protected behind the clavicle, is more likely to be injured by penetrating trauma than blunt injuries because of its location and structural characteristics.^[1,2,7,10]^ Blunt injury of the SCA can occur due to a sudden decrease in speed (commonly caused by motor vehicle accidents or falls^[2]^) that affects the neck, chest, and upper limbs. The evoked force is transmitted to fixed points along the blood vessel, typically at the origin of the vertebral and internal thoracic arteries, detaching the subclavian vessel.^[8]^ Furthermore, a bone fragment produced by a fractured first rib or clavicle can cause laceration of the SCA.^[8]^

The characteristic signs of SCA injury include a decrease or absence of radial or ulnar pulses on the injured side, hand ischemia, the presence of an expanding or pulsating hematoma above or below the clavicle, and an overlying bruise.^[2]^

In a penetrating SCA injury, direct compression using a Foley catheter may be effective in stopping bleeding. On the other hand, however, direct compression is not effective in achieving hemostasis in a blunt injury because of the surrounding bony structures and muscles. Moreover, the longer the bleeding time, the higher the chances of disseminated intravascular coagulation (DIC) and the higher the mortality. In addition, because injury to the SCA in blunt trauma is usually accompanied by damage to bone, muscle, or the brachial plexus, bleeding control through direct compression may cause extra damage.^[1,2,10]^ Therefore, it is important to find a way to shorten the bleeding time as much as possible without causing additional damage to the surrounding tissue.

CT angiography can be a reliable modality for diagnosis and can help plan the operative approach.^[6,10]^ Duplex ultrasonography also provides crucial information in hemodynamically unstable patients and allows careful selection of candidates indicated for surgical intervention.^[10]^ Patients with low-grade SCA injury without signs of limb ischemia can be managed by close observation with serial CT angiography or ultrasonography, along with anticoagulation or antiplatelet agents.^[11]^ Conversely, nonoperative management of high-grade SCA injury is associated with poor outcomes; therefore, it is essential to consider either an open or an endovascular approach.^[12]^

Traditionally, open surgical repair of the SCA has been considered the standard treatment option in case of injury.^[1,13]^ Open surgical methods require an incision in the skin and dissection of the injured area to locate the artery or to cut off blood flow proximal to the injured area to prevent further bleeding, which in some cases even requires a median sternotomy or cutting off the clavicle.^[1,2,4,5]^ Sometimes it requires an extensive incision to obtain proximal and distal control, which is invasive, difficult to perform, and associated with high morbidity.^[8,14]^

In contrast, the endovascular approach helps locate the ruptured site easily and control the active bleeding using a covered stent graft, thereby reducing blood loss, preventing the development of DIC, and improving mortality and morbidity of SCA injuries. In previous studies, the endovascular technique was found to be less invasive, resulting in lesser blood loss, reduced need for RBC transfusion, and shorter length of hospital stay compared with an open surgical approach.^[4,10]^

The endovascular technique is effective if the blood vessel is partially damaged but may be difficult if the blood vessel is transected.^[13]^ In this case, if the transected segment is not very long, hemostasis and recanalization of blood flow through a stent graft are possible through the rendezvous technique.^[4,9]^ The use of stent grafts is, however, limited by the location of the injury in the SCA. Stent grafts cannot be placed in areas where the distal end of the graft is outside the thoracic cage or where blood vessels are compressed.^[5]^ If the transected segment is long or a stent graft is not available and conversion to the open technique is necessary, balloon clamping of the proximal site can be used to stop bleeding instead of vessel ligation.^[6]^

In both of our cases, the endovascular procedure took less than an hour and we can minimize blood loss, which prevented the development of critical complications, such as DIC. In the second patient, the situation required prompt treatment of the arterial injury and damage to the duodenum and liver. The endovascular technique saved substantial time, allowing us to start the abdominal damage control surgery quickly, and the patient was discharged in a better state.

## 4. Conclusion

In this study, we confirmed the efficiency of endovascular technique through the case of a patient with SCA injury caused by 2 different mechanisms. Rapid diagnosis and appropriate treatment using endovascular technique saved both the patient’s life and arm. Furthermore, this study confirms that endovascular techniques can be utilized not only for diagnosis and definitive treatment but also as bridging therapy in patients with SCA injuries. These findings suggest that endovascular approaches can be considered as a first-line therapy for managing SCA injuries.

## Acknowledgments

The authors would like to thank the patient and his family for the informed written consent for publication of this case report and accompanying image.

## Author contributions

**Writing – original draft:** Gun Woo Kim.

**Supervision:** Sung Hoon Cho, Kyoung Hoon Lim.

**Writing – review & editing:** Kyoung Hoon Lim, Suyeong Hwang.
